# Bacterial Cellulose/Cellulose Imidazolium Bio-Hybrid Membranes for In Vitro and Antimicrobial Applications

**DOI:** 10.3390/jfb14020060

**Published:** 2023-01-20

**Authors:** Ahmed Salama, Ahmed K. Saleh, Iriczalli Cruz-Maya, Vincenzo Guarino

**Affiliations:** 1 Cellulose and Paper Department, National Research Centre, 33 El-Bohouth St., Dokki, Giza P.O. Box 12622, Egypt; 2 Institute of Polymers, Composites and Biomaterials, National Research Council of Italy, Mostra d’Oltremare, Pad.20, V.le J.F. Kennedy 54, 80125 Naples, Italy

**Keywords:** bacterial cellulose, cationic cellulose, bio-composites, cytocompatibility, antimicrobial response

## Abstract

In biomedical applications, bacterial cellulose (BC) is widely used because of its cytocompatibility, high mechanical properties, and ultrafine nanofibrillar structure. However, biomedical use of neat BC is often limited due to its lack of antimicrobial properties. In the current article, we proposed a novel technique for preparing cationic BC hydrogel through in situ incorporation of cationic water-soluble cellulose derivative, cellulose bearing imidazolium tosylate function group (Cell-IMD), in the media used for BC preparation. Different concentrations of cationic cellulose derivative (2, 4, and 6%) were embedded into a highly inter-twined BC nanofibrillar network through the in situ biosynthesis until forming cationic cellulose gels. Cationic functionalization was deeply examined by the Fourier transform infrared (FT–IR), NMR spectroscopy, scanning electron microscopy (SEM), energy-dispersive X-ray spectroscopy (EDS), and X-ray diffraction (XRD) methods. In vitro studies with L929 cells confirmed a good cytocompatibility of BC/cationic cellulose derivatives, and a significant increase in cell proliferation after 7 days, in the case of BC/Cell-IMD3 groups. Finally, antimicrobial assessment against *Staphylococcus aureus*, *Streptococcus mutans*, and *Candida albicans* was assessed, recording a good sensitivity in the case of the higher concentration of the cationic cellulose derivative. All the results suggest a promising use of cationic hybrid materials for biomedical and bio-sustainable applications (i.e., food packaging).

## 1. Introduction

Sustainable and multifunctional materials derived from renewable resources are increasingly in demand in modern society [1,2,3,4,5]. The development of biobased polymers, and particularly biobased polysaccharides, with antimicrobial activity has been studied in various areas of application, such as medicine, pharmacy, and food [6]. As a result of its high hydroxyl content, cellulose represents an excellent resource for producing multifunctional materials [7,8]. Cellulose, the most abundant organic polymer, is a linear homopolysaccharide containing units of D-anhydroglucopyranose, connected by β(1→4) glycosidic links [9]. Anhydroglucopyranose units contain three hydroxyl groups that form strong intra- and intermolecular hydrogen bonds to form fibrils that have crystalline and amorphous regions [10]. The multiscale hierarchical structure arising from the self-assembly of nanocelluloses into higher-order structures is accompanied with presenting low density, high mechanical strength, and good flexibility and thermal stability [11]. There has been extensive research on the biomedical and wound-healing applications of nanocelluloses [12]. Cellulose nanocrystals and cellulose nanofibrils can be isolated from biomass to make nanoscale cellulose, which displays exciting properties, such as low toxicity, being lightweight, and a high specific surface area, and can be produced at an industrial scale [13,14]. Among the reported cellulose nanoparticles, BC was reported as promising biomaterial with high purity. The BC network consists of three-dimensional nanofibers that are formed by particular sorts of bacteria, such as *Gluconacetobacter, Agrobacterium*, and *Sarcina Gluconobacter xylinus* [15]. Several factors make BC more appealing than plant cellulose, including its high crystallinity, high purity, high water retention, remarkable mechanical properties, and improved cytocompatibility [16]. As a result, it has several potential applications, especially in biocatalytic, biomedical, and pharmaceutical fields [17,18]. BC lacks certain properties that are essential for biomedical applications, such as antibacterial and antioxidant properties. Consequently, BC composite materials have been developed using bioactive polymers, nanomaterials, and solid particles to expand their uses. For example, a semi-interpenetrating hydrogel consisting of BC and chitosan can be formed under glutaraldehyde cross-linking, and the antibacterial ability is determined by the amount of chitosan [19]. Tatyane Duran Lopesa et al. studied the production of bacterial cellulose/hyaluronic acid hybrid membranes, under static conditions after three distinct time points. The results proved that the crystal structure, surface roughness, thermal stability, and hydrophobic/hydrophilic character of bacterial cellulose/hyaluronic acid hybrid membranes changed [20]. A bio-hybrid system based on BC and metal–phenolic networks has been developed by Shuo Tang et al. An antimicrobial effect was conferred upon BC nanofibrillar networks by complexing and embedding plant-derived tannic acid with various metal ions. During the preparation of cationized BC, tannic acid bridged between quaternary ammonium salt and BC. Cationic BC@metal–phenolic network composites exhibited excellent inhibitory effects on the growing of *Escherichia coli* and *Staphylococcus aureus*. The food packaging and biomedical industries may benefit from this antimicrobial BC/metal–phenolic network material [21]. It has been reported that polysaccharides with cationic functional groups are effective antimicrobial agents [22]. The construction of scaffolds from functionalized natural polysaccharides can impart the inherent bioactivity of BC such as antimicrobial properties.

Herein, a new procedure is proposed based on the incorporation of cationic cellulose derivatives in BC membranes during the fermentation process. The work is aimed at investigating the in situ BC crystallization, morphology, thermal stability, in vitro cytocompatibility, and antimicrobial properties. The proposed study paves the way for the development of novel composite materials through a simple, rapid, and economic technique, without the addition of any components, in order to use them as bio- based materials for different applications (i.e., wound healing, drug delivery, food packaging).

## 2. Materials and Methods

### 2.1. Materials

Microcrystalline cellulose, p-toluenesulfonyl chloride and 1-methylimidazole were provided by Sigma-Aldrich. The ionic liquid, 1-Butyl-3-methylimidazolium chloride was purchased from IoLiTec, Heilbronn, Germany. The rest of the chemicals are analytical grade and are not further purified.

### 2.2. Synthesis

#### 2.2.1. Tosyl Cellulose Preparation

Tosyl cellulose was prepared according to our previous study [23]. In brief, 2.0 g of cellulose was dissolved in 18 g ionic liquid, 1-Butyl-3-methylimidazolium chloride, at 80 °C for 10 h. Then, the obtained solution was cooled to 25 °C and 10 mL pyridine was mixed with the solution. Then, 7.06 g *p*-toluenesulfonyl was dissolved in 10 mL pyridine and added dropwise to the cellulose/ionic liquid/pyridine. After stirring the reaction mixture for 12 h, it was precipitated in ethanol. The white precipitate was separated and washed with ethanol and dried in vacuum at 60 °C.

#### 2.2.2. Preparation of Cellulose Containing Imidazolium Tosylate

In 5 mL DMSO, we dissolved 1 g of tosyl cellulose, and then added 3.1 g of methyl imidazole (3.8 mmol). After stirring at 100 °C overnight, the reaction mixture was cooled to room temperature and poured into 1 mm mL of ethyl acetate. After separation, the precipitate was washed with ethyl acetate several times (20 mL each), and then dried in vacuum at 70 °C. The yield of cellulose containing imidazolium tosylate was 3.1 g. Elemental composition: C 4.8%, H 4.5%, N 5.5%, S 4.9%.

#### 2.2.3. Characterization Methods

Elemental analysis was performed via Elemental Vario Micro Cube apparatus. The attenuated total reflectance Fourier transform infrared (ATR-FTIR) spectra were attained using a Thermo Nicolet FTIR. Liquid NMR spectroscope. ^13^C NMR spectra in solution were recorded on a Bruker Avance 400 spectrometer. X-ray diffraction measurements were achieved with Empyrean PANalytical diffractometer utilizing a Cu Kα1 radiation (λ = 1.54056 Å).

### 2.3. Preinoculum Preparation

Lactiplantibacillus plantarum AS.6 (L plantarum AS.6) was isolated previously as a BC producer strain [24]. Hestrin and Schramm (HS) media adjusted to contain 2% glucose, 0.5% (*w*/*v*) yeast extract, 0.5% (*w*/*v*) peptone, 0.27% (*w*/*v*) disodium hydrogen phosphate, 0.115% (*w*/*v*) citric acid, and 0.5% (mL/v) ethanol at pH 5.5 and incubated at 30 °C for 2 days under agitated conditions (200 rpm) was inoculated with a single colony of L plantarum AS.6 for preinoculum preparation [25].

### 2.4. In Situ Preparation of BC/Cellulose Imidazolium (BC/Cell-IMD) Hydrogel

**Figure 1:**
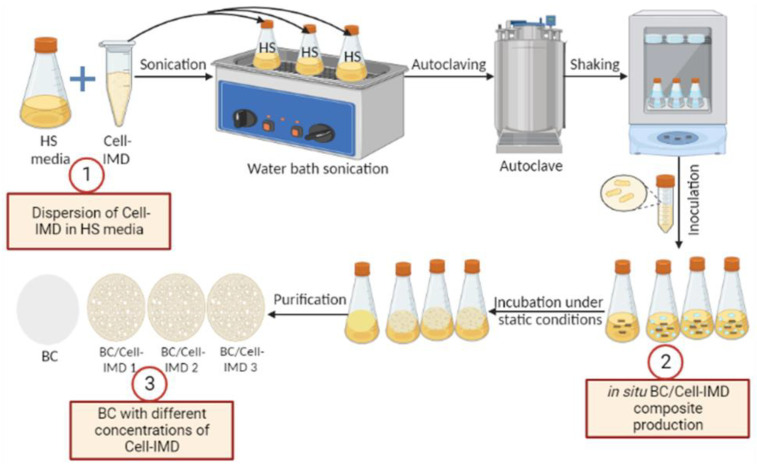
Schematic description of the in situ preparation of BC/Cell-IMD hybrids.

Cell-IMD was evaluated for in situ preparation of BC hydrogel by using L plantarum AS.6 under static conditions in accordance with the method of G. Gao et al. [26] with some modifications as illustrated in Figure 1. For production of BC/Cell-IMD hydrogel, the HS media supplemented with different concentrations of BC/Cell-IMD (0.2, 0.4, and 0.6% (*w*/*v*)) was first dispersed by using a very mild water bath sonication (720 W, 50/60 Hz, 230 V, Spain) at 60 °C for 2 h and then shaken at 200 rpm for 24 h to ensure the complete dispersion of Cell-IMD. Then, the media pH was adjusted to 6 and autoclaved at 121 °C for 20 min and left to cool, and then inoculated by 10% of the preinoculum and then incubated for 7 days at 30 °C under static conditions. The production of BC free from Cell-IMD (control) was also produced using the same method without the addition of Cell-IMD under static conditions. The generated hydrogels are named BC, BC/Cell-IMD1, BC/Cell-IMD2, and BC/Cell-IMD3. Afterwards, the obtained BC and BC/Cell-IMD hydrogels were washed several times by distilled water to get rid of the residue medium composition. The BC samples were then soaked in 0.5% NaOH at 90 °C for 30 min to remove bacterial contaminants and other metabolites integrated on the BC samples, followed by deionized water washing until a neutral pH was obtained [27].

### 2.5. Cytocompatibility Assays

As for cytocompatibility tests, fibroblasts derived from mouse (L929, Sigma Aldrich, Saint Louis, MO, USA) cells were cultured in a 75 cm^2^ cell culture flask in Dulbecco’s (DMEM) supplemented with 10% of fetal bovine serum (FBS), antibiotic solution (streptomycin 100 µg/mL and penicillin 100 U/mL) and 2 mM of L-glutamine, incubated at standard conditions (37 °C in a humidified atmosphere with 5% CO_2_ and 95% air). Before the assays, samples were cut (Ø 6 mm) and placed in a 96-well cell culture plate, washed with PBS, and sterilized 30 min with a solution with 70% of ethanol. Then, samples were washed three times and dried under the hood.

L929 cells (5 × 10^3^ as seeding density per well) were incubated with supplemented DMEM under standard conditions. For in vitro tests, The Cell Proliferation Kit II (XTT, Sigma Aldrich, Italy) was used. This colorimetric assay is based on the reduction of a yellow tetrazolium salt (sodium 3’-[1- (phenylaminocarbonyl)- 3,4- tetrazolium]-bis (4-methoxy6-nitro) benzene sulfonic acid hydrate or XTT) to an orange formazan dye by metabolically active cells. For cell adhesion, after 12 and 24 h, samples were washed two times to remove the unattached cells and a solution of medium with XTT was added to incubate for four hours. After the incubation time, the supernatant was recovered and placed in a 96-well plate reader. Absorbance measurements were recorded at 450 nm. Results of cell adhesion are presented as percentage of adhesion with respect to the tissue culture plate (TCP).

For cell viability, 1, 3, and 7 days were considered. After the incubation time, cell culture media was removed and changed by fresh media containing XTT working solution, according to the manufacturer instructions. After four hours of incubations, the supernatant was recovered and placed in a 96-well plate reader to measure the absorbance at 450 nm. The increase in absorbance is related to the increase in the activity of mitochondrial dehydrogenases, thus, the living cells. Results were represented as mean ± standard deviation (*n* = 3). Analysis of variance (ANOVA) with Tukey’s post hoc test was used to detect differences between groups. A value of *p* < 0.05 was considered to determine statistically significant differences.

### 2.6. Antimicrobial Evaluation of BC and BC/Cell-IMD Hybrids

The antimicrobial assessment of the prepared hydrogels was evaluated against five selected pathogens, including *Escherichia coli* American Type Culture Collection (ATCC) 25,922 (*E. coli*), *Salmonella typhimurium* ATCC 14,028 (*S. typhimurium*) as Gram-negative bacteria; *Staphylococcus aureus* ATCC 25,923 *(S. aureus*) and *Streptococcus mutans* ATCC 25,175 (*S. mutans*) as Gram-positive bacteria; and *Candida albicans* ATCC 10,231 (*C. albicans*) as a yeast model. The microbial strains were cultivated in test tube containing 5 mL of Mueller Hinton broth composed of 0.2% (*w*/*v*) beef extract, 1.75% (*w*/*v*) acid hydrolysate of casein, and 0.15% (*w*/*v*) starch, under shaking cultivation (200 rpm) for one day at 30 °C. The antimicrobial activity of the generated BC and BC/Cell-IMD hydrogels was evaluated through disk diffusion with the determination of inhibition zones in accordance with the method described in previous work with some modifications [28]. In brief, 100 µL of serially diluted pathogens (10^8^ CFU/mL) was separately distributed on petri dishes of Mueller Hinton (20 g/L agar) media, along with disks (3 mm in diameter) of the BC/Cell-IMD hydrogel at different concentrations of Cell-IMD. BC hydrogel on its own was used as a negative control. At 30 °C for one day, the plates were incubated, where the antibacterial activity was assessed by measuring the developed inhibition zone diameter (including the hydrogel disk) after incubation. All hydrogels were sterilized for 30 min under UV light before application to ensure aseptic conditions. All experiments were repeated three times, and the mean result was represented.

## 3. Results and Discussion

### 3.1. Preparation of Cationic Cellulose Containing Imidazolium Tosylate

**Figure 2:**
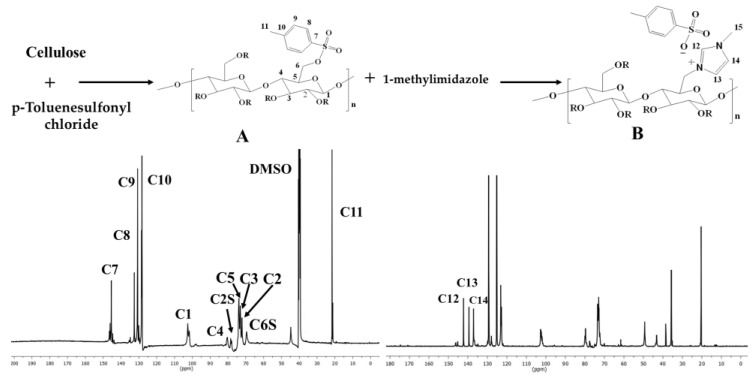
Synthesis scheme and ^13^C NMR spectroscopy for tosyl cellulose (**A**) and cationic cellulose containing imidazolium tosylate (**B**).

Cationic water-soluble cellulose derivative holding imidazolium tosylate was produced through direct reaction between tosyl cellulose and 1-methylimidazole. The formation of cationic cellulose was proved by various analytical methods including elemental analysis and ^13^C NMR. Elemental analysis recorded for cellulose was C 42.9% and H 6.4%, while tosyl cellulose exhibited C 45.3%, H 5.2%, and S 10.2%. The appearance of sulfur confirms the tosylation process. However, the new cationic cellulose containing imidazolium group exhibited C 44.8%, H 4.5%, N 5.5%, and S 4.9%. NMR spectroscopy was used to demonstrate the preparation of cationic cellulose, and the spectra are shown in Figure 2.

Tosyl cellulose displays aromatic carbon signals at 145.6, 132.5, 130.7, and 128.1. The signal at 103.3 corresponds to C1 in the anhydroglucose unit. Moreover, anhydroglucose carbon centers are characterized by signals between 80.7 and 73.5 ppm. It is possible that the signal at 68.8 ppm refers to tosylated C6, whereas the signal at 60.7 ppm is due to nontosylated C6. The signal assigned to C11 carbon (CH_3_) is at 21.1. Cationic cellulose with imidazolium tosylate has a signal at 36.1, which confirms the formation of N-CH_3_. In addition, the imidazolium group carbons are responsible for new signals at 137, 140, and 142 ppm. It appears that imidazolium tosylate was formed on cellulose according to ^13^C NMR spectroscopy.

**Figure 3:**
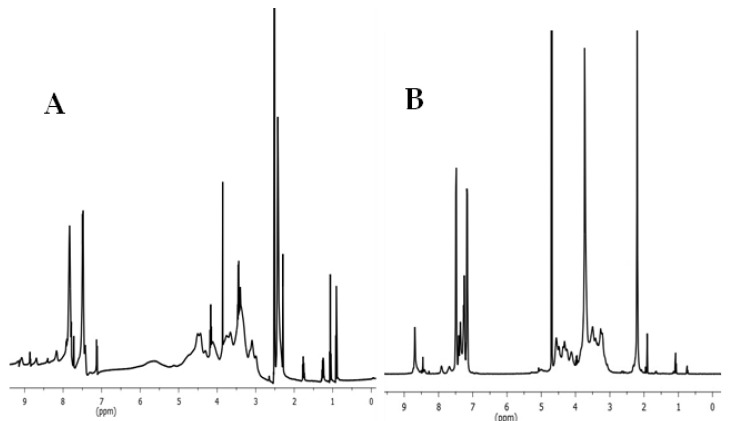
(**A**) ^1^HNMR spectrum of tosyl cellulose (solvent: DMSO-d6). (**B**) ^1^HNMR spectrum of cationic starch containing imidazolium tosylate (solvent: D_2_O).

The ^1^H NMR spectra of tosyl cellulose and cellulose containing methylimidazolium cationic groups are displayed in Figure 3. As can be seen in the ^1^H NMR spectrum of tosyl cellulose, proton resonances of anhydroglucose units are found at 3.1–4.7 ppm. The peak at 7.4–7.9 ppm refers to the phenyl protons of the tosyl group. Additionally, protons from the CH_3_ of the tosyl group confirm the peak at 2.3. However, after nucleophilic substitution of the tosyl group with the methylimidazolium group, new signals appeared. The functionalization of cellulose is further confirmed by the presence of the protons of imidazolium tosylate at 7.3, 7.4, and 8.7 ppm. The new signal at low frequency may refer to the hydrogen of the methyl group, which demonstrates that the methyl imidazole has been successfully formed.

### 3.2. BC/Cell-IMD Bio-Hybrids’ Characterization

Dry purified BC was analyzed for its elemental composition using elemental analysis data as follow: C 45.6%; H 6.8%; N 1.8%: S 0.5%. The results of this study are consistent with the literature [29]. The presence of nitrogen and sulfur in the BC sample, most likely, is caused by the remnants of bacterial cells that have been destroyed [30]. Elemental analysis data for nitrogen content in dry BC/Cell-IMD1, BC/Cell-IMD2, and BC/Cell-IMD3 hybrids showed considerable increase and recorded 5, 6.5, and 7.3%, respectively. Moreover, the sulfur content in dry BC/Cell-IMD1, BC/Cell-IMD2, and BC/Cell-IMD3 was slightly increased and recorded 1.1, 1.7, and 2.3%, respectively. These results are good indications for the successful formation of BC/Cell-IMD hybrids.

**Figure 4:**
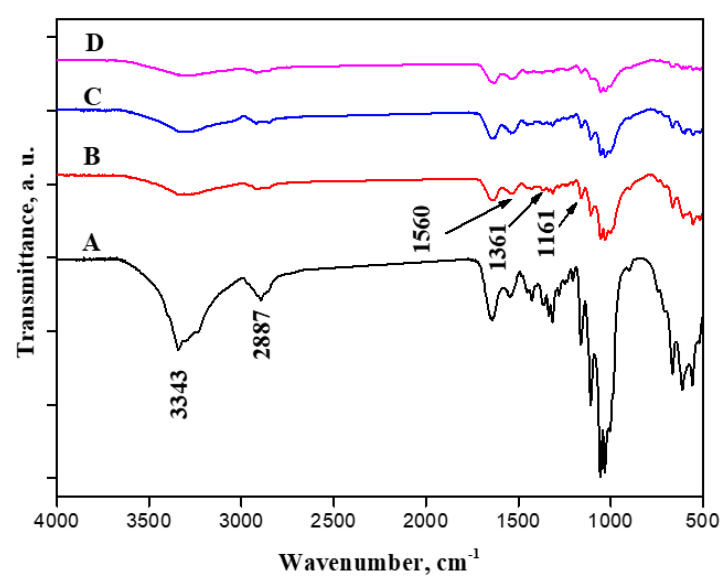
FTIR spectroscopy of BC (**A**), BC/Cell-IMD1 (**B**), BC/Cell-IMD2 (**C**) BC/Cell-IMD3 (**D**).

ATR-FTIR spectroscopy was utilized to distinguish BC and BC containing cationic cellulose (BC/Cell-IMD) at different concentrations. Line A in Figure 4 illustrates characteristic vibration bands of bacterial cellulose. The characteristic peak at 3334 cm^−1^ is related to hydroxyl groups and inter- and intramolecular hydrogen bonds [31]. Additionally, peaks near 2887 cm^−1^ are associated with the C-H stretching of CH_2_ and CH_3_ groups [32,33]. Cationic cellulose peaks were observed after the dissolution of cationic cellulose in BC preparation media during the fermentation process. The typical bands of the tosyl group at 1161 cm^−1^ (υs SO_2_), 1361 cm^−1^ (υas SO_2_), and 1539 cm^−1^ (ν C = Caromatic) were observed, which suggests the presence of tosylate groups as counter anions [34]. Furthermore, the absorption band around 3400 cm^−1^ was reduced, suggesting a decrease in hydroxyl group intensity. It is possible that the peak around 1560 cm^−1^ became more intense and broader because of the presence of the C-N stretch characteristic of methylimidazolium.

**Figure 5:**
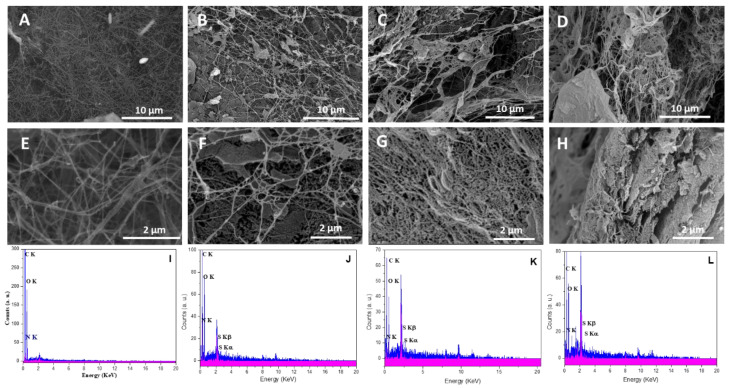
Morphological characterization of BC/Cell-IMD hybrids: SEM images for BC (**A**,**E**), BC/Cell-IMD1 (**B**,**F**), BC/Cell-IMD2 (**C**,**G**), BC/Cell-IMD3 (**D**,**H**); EDX results for BC (**I**), BC/Cell-IMD1 (**J**), BC/Cell-IMD2 (**K**), BC/Cell-IMD3 (**L**).

The surface morphologies of neat BC and BC holding various concentrations of cationic cellulose were observed using SEM analysis (Figure 5). BC containing low cationic cellulose concentrations, BC/Cell-IMD1, still maintained the original surface fiber morphology. However, after incorporating a high concentration of cationic cellulose, the morphology of bacterial cellulose was significantly changed to more densely packed and interconnected fibers, BC/Cell-IMD2 and BC/Cell-IMD3. Moreover, the BC formed in the presence of high concentrations of cationic cellulose showed a porous structure under high magnification, possibly ascribed to the weakening of the hydrogen bonding strength due to the incorporation of the cationic cellulose bearing imidazolium group. Another possible reason is that the incorporation of highly substituted cationic cellulose imparts a large amount of cationic charges on the surface of BC, which induce electrostatic repulsion between the particles. To further reveal the successful BC hyybrids, energy-dispersive X-ray (EDX) was performed (Figure 5). Nitrogen and sulfur elements presented in the cationic cellulose were found, which indicate that cationic cellulose was successfully incorporated in the BC network. In fact, BC formation is hardly affected unless interfering additives are incorporated into the culture medium.

**Figure 6:**
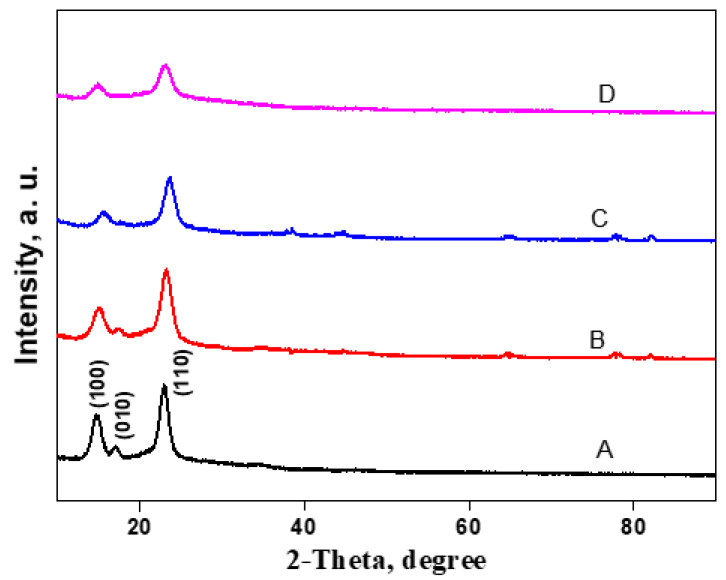
XRD analysis of BC/Cell-IMD hybrids: spectra of BC (**A**), BC/Cell-IMD1 (**B**), BC/Cell-IMD2 (**C**), BC/Cell-IMD3 (**D**).

The impact of cationic cellulose incorporation on the BC crystalline structure was studied by X-ray diffraction (XRD) (Figure 6). The XRD diffraction pattern of BC exhibited three main cellulose typical peaks at 2θ = 14.6°, 16.9°, and 22.8°, which were assigned to (100), (010), and (110) crystallographic planes, respectively. In comparison to the peak at 2θ = 16.7°, the peak at 2θ = 14.5° had a much higher strength, suggesting that cellulose formed by *Gluconacetobacter hanseii* contained a predominant cellulose Iα allomorph. The BC/Cell-IMD films show similar XRD patterns as BC, indicating that the incorporation of cationic cellulose in a certain amount could maintain the crystalline structure of BC.

### 3.3. In Vitro Studies: Cytocompatibility

**Figure 7:**
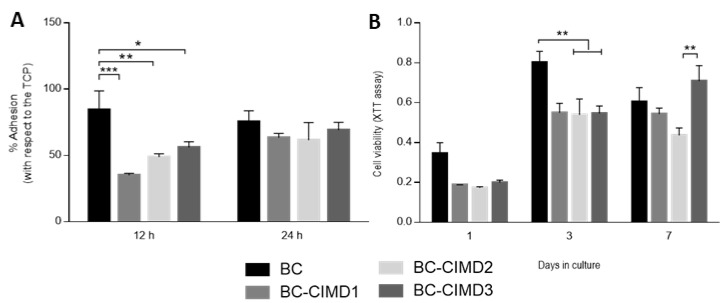
In vitro response of L929 cells onto BC/Cell-IMD hybrids: (**A**) adhesion and (**B**) viability tests. (* *p* < 0.05; ** *p* < 0.01; *** *p* < 0.001).

BC is an attractive material for its use as biomaterial due to its good cytocompatibility and fibrous structure, similar to the natural extracellular matrix [35]. Cell adhesion was measured after 12 and 24 h (Figure 7A). After 12 h, the control of BC presented a better cell adhesion with respect to the other groups, and similar behavior with respect to the control (TCP). In the case of the BC/Cell-IMD1, BC/Cell-IMD2, and BC/Cell-IMD3 groups, cell adhesion was significantly lower than BC, increasing after 24 h until reaching a similar behavior to BC. Moreover, it has been reported that the modification of the cellulose surface (cationic cellulose) improves the cell attachment, without the use of additional modifications as in the case of other natural or synthetic polymers [36].

The favorable conditions provided by cellulose-based materials for adhesion, proliferation, and cell differentiation have been studied for a wide variety of applications [37]. The cell viability onto BC and BC/Cell-IMD at different concentrations is reported in Figure 7B At the first and third day, the proliferation rate was similar for all groups with cationic cellulose, while BC showed an increase in cell proliferation. Bacterial cellulose has been demonstrated to be cytocompatible without inflammatory reactions [38,39]; in addition, the possibility of modifying or combining BC increases the research attention for its use in the field of biomaterials with good cytocompatibility. Indeed, the electrostatic interactions between the material charge and the surrounding environment (i.e., proteins) influence the adsorption of proteins; thus, the cell adhesion and proliferation [40,41]. For instance, in the results at seven days, the presence of a higher concentration of Cell-IMD shows a significant increase in cell viability that can be ascribable to the more dense and interconnected fibers due to the high concentration of cationic cellulose. Moreover, the decrease in crystallinity may allow for a better proliferation as in the case of amorphous polymers [42,43].

### 3.4. Antimicrobial Studies

**Figure 8:**
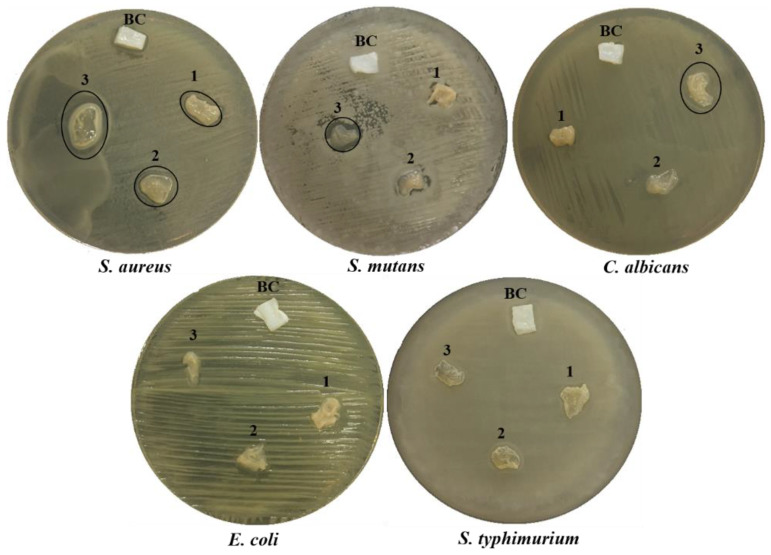
Antimicrobial activity expressed as halo zones of BC and BC/Cell-IMD hydrogel at different concentrations of BC/Cell-IMD1, BC/Cell-IMD2, and BC/Cell-IMD3 represents 1, 2, and 3, respectively, against five pathogenic microbes.

The antimicrobial performance of the BC and BC/Cell-IMD hydrogel was investigated by the agar disc diffusion method. Notably, various concentrations were initially considered to perform a preliminary evaluation of the significance of antimicrobial data. Accordingly, it was verified that there was no significant antimicrobial response below 0.2% (data not shown). Hence, only the data collected for concentrations ranging from 0.2 to 0.6% were reported in Table 1. In agreement with the reported data, Figure 8 confirmed considerable inhibition zones for the BC/Cell-IMD hydrogels against all pathogens tested except Gram-negative bacteria. According to the diameter of the inhibition zone observed, the antimicrobial activity was enhanced as the concentration of BC/Cell-IMD was increased. Furthermore, the sensitivity of Gram-positive bacteria against BC/Cell-IMD is higher than that observed against Gram-negative bacteria. These results agree with other studies [44,45]. Therefore, these BC/Cell-IMD hydrogels are believed to have great potential for use as antimicrobial agents.

The difference in the antimicrobial behavior of the BC/Cell-IMD could be due to the different cell walls. The results showed high antimicrobial properties for BC/Cell-IMD, which may be due to the presence of imidazolium tosylate. In the alkyl chain of imidazolium, there are more than ten carbon atoms, especially C12 and C14, which are responsible for activity against a wider range of Gram-positive than Gram-negative bacteria [46]. The activity of imidazolium against bacteria is enhanced by increasing the alkyl chain length (carbon atoms), as reported in previous studies [47,48]. Another study reported that the antimicrobial activity of the imidazolium group is related to the presence of the hydroxyethyl chain and the methyl group in the imidazolium ring structure [48]. Conversely, the potential electrostatic interaction between the imidazolium cations and the negatively charged cell wall is the main antimicrobial force [49,50].

From our results, the effect of the pure BC hydrogel against all microbial pathogens was negligible, in agreement with our previous reported studies [51]. However, promising trials have been carried out to enhance the antimicrobial properties of BC. For example, the BC sample was functionalized via in situ or ex situ modifications in order to create an active BC hydrogel as an antimicrobial agent, so that several studies were carried out to introduce antimicrobial properties into BC; for example, Jorge et al. reported that the BC was functionalized by bovine lactoferrin, and this composite showed bactericidal efficiency against two food pathogens, *E coli* and *S aureus* [52]. The BC membrane and magnesium oxide nanoparticles were fabricated by in situ and ex situ modifications, and the antibacterial activity of the ex situ synthesized nanohybrid against *S. aureus* and *E. coli* bacteria was greater than that of both in situ synthesized samples [45]. The nanocomposite based on BC/graphene oxide/copper oxide nanoparticles displayed better antibacterial activity against Gram-positive (*S. aureus* and *B. subtilis*) than Gram-negative (*E. coli* and *P. aeruginosa*) bacteria [53]. By using biological self-generation and in situ reduction, the new composite was prepared from BC/chitosan/polydopamine/silver nanoparticles. The composite demonstrated broad-spectrum inhibition of *E. coli*, *S. aureus*, and *P. aeruginosa*, and this inhibition was maintained at more than 80% after 48 h of continuous use [54]. The BC hydrogel was loaded with pomegranate peels extract by ex situ modification and the new composite membrane revealed significant broad-spectrum antimicrobial activity [24].

The antimicrobial properties of cationic cellulose depend on the imidazolium-containing cationic alkyl chain. The incorporation of hydroxyethyl and methyl groups (in the ring at two and three centers) is critical to its antimicrobial activity [48]. Among the most cited and proposed mechanisms for action of cationic polymers is their interaction with the outer surface of bacteria and fungi, followed by an electrostatic attraction culminating in surface rupture that releases intracellular components [55].

## 4. Conclusions

Bacterial cellulose/cationic cellulose containing imidazolium hybrid membranes were created in situ, through the addition of cationic cellulose in the fermentation process. The concentration of cellulose containing imidazolium during the fermentation process impacts the main properties of the obtained membranes. Morphological and Elemental analyses (SEM/EDX) confirmed the presence of nitrogen content in the membrane, thus confirming the hybrid formation. Moreover, XRD results exhibited a decrease in the crystallinity of the hybrids compared with BC. The crystallinity of the film seems to be affected by the addition of Cell-IMD at different concentrations. The Gram-positive bacteria and Candida albicans showed more sensitivity to BC/Cell-IMD, while the Gram-negative bacteria showed more resistance to the nanocomposite hydrogel. The use of hybrid membranes of BC and Cell-IMD could be a promising strategy for a wide range of biomedical applications due to the intrinsic antibacterial properties of Cell-IMD and good cytocompatibility of BC.

## Figures and Tables

**Table 1:** The antimicrobial activity of the BC and BC/Cell-IMD hydrogel with different concentrations of Cell-IMD against five pathogenic microorganisms.

Organisms	Diameters of Inhibition Zone (mm)			
	BC	BC/Cell-IMD		
		0.2%	0.4%	0.6%
*E. coli*	0.0 ± 0.0	0.0 ± 0.0	0.0 ± 0.0	0.0 ± 0.0
*S. typhimurium*	0.0 ± 0.0	0.0 ± 0.0	0.0 ± 0.0	0.0 ± 0.0
*S. aureus*	0.0 ± 0.0	7 ± 2.09 ^a^	12 ± 2.25 ^a^	17 ± 2.47 ^a^
*S. mutans*	0.0 ± 0.0	0.0 ± 0.0	0.0 ± 0.0	11 ± 1.78 ^c^
*C. albicans*	0.0 ± 0.0	0.0 ± 0.0	0.0 ± 0.0	15 ± 0.83 ^b^

Statistical significance was indicated alphabetically (in descending significance order where a > b > c, etc.) according to ANOVA analysis with CoStat program using least significant difference test (LSD) at *p*-value ≤ 0.05.

## Data Availability

Not applicable.
